# Deployment of machine learning algorithms to predict sepsis: systematic review and application of the SALIENT clinical AI implementation framework

**DOI:** 10.1093/jamia/ocad075

**Published:** 2023-05-12

**Authors:** Anton H van der Vegt, Ian A Scott, Krishna Dermawan, Rudolf J Schnetler, Vikrant R Kalke, Paul J Lane

**Affiliations:** Queensland Digital Health Centre, The University of Queensland, Brisbane, Queensland, Australia; Department of Internal Medicine and Clinical Epidemiology, Princess Alexandra Hospital, Brisbane, Australia; Centre for Information Resilience, The University of Queensland, St Lucia, Australia; School of Information Technology and Electrical Engineering, The University of Queensland, St Lucia, Australia; Patient Safety and Quality, Clinical Excellence Queensland, Queensland Health, Brisbane, Australia; Safety Quality & Innovation, The Prince Charles Hospital, Queensland Health, Brisbane, Australia

**Keywords:** sepsis prediction, systematic review, AI implementation, machine learning, artificial intelligence

## Abstract

**Objective:**

To retrieve and appraise studies of deployed artificial intelligence (AI)-based sepsis prediction algorithms using systematic methods, identify implementation barriers, enablers, and key decisions and then map these to a novel end-to-end clinical AI implementation framework.

**Materials and Methods:**

Systematically review studies of clinically applied AI-based sepsis prediction algorithms in regard to methodological quality, deployment and evaluation methods, and outcomes. Identify contextual factors that influence implementation and map these factors to the SALIENT implementation framework.

**Results:**

The review identified 30 articles of algorithms applied in adult hospital settings, with 5 studies reporting significantly decreased mortality post-implementation. Eight groups of algorithms were identified, each sharing a common algorithm. We identified 14 barriers, 26 enablers, and 22 decision points which were able to be mapped to the 5 stages of the SALIENT implementation framework.

**Discussion:**

Empirical studies of deployed sepsis prediction algorithms demonstrate their potential for improving care and reducing mortality but reveal persisting gaps in existing implementation guidance. In the examined publications, key decision points reflecting real-word implementation experience could be mapped to the SALIENT framework and, as these decision points appear to be AI-task agnostic, this framework may also be applicable to non-sepsis algorithms. The mapping clarified where and when barriers, enablers, and key decisions arise within the end-to-end AI implementation process.

**Conclusions:**

A systematic review of real-world implementation studies of sepsis prediction algorithms was used to validate an end-to-end staged implementation framework that has the ability to account for key factors that warrant attention in ensuring successful deployment, and which extends on previous AI implementation frameworks.

## INTRODUCTION

Sepsis accounts for nearly 20% of deaths worldwide, killing over 11 million people in 2017.^1^ Sepsis has been defined as a “life-threatening organ dysfunction caused by a dysregulated host response to infection”.^2^^,^^3^ Early recognition and treatment of sepsis can reduce mortality, and rule-based surveillance systems for detecting sepsis in hospital settings can improve outcomes.^4^^,^^5^

More recently, sepsis prediction algorithms employing artificial intelligence (AI),^6–8^ herein called machine learning algorithms (MLAs), that can detect evolving sepsis in patients earlier than rule-based methods, have proliferated.^9^^,^^10^ Most MLA studies assess performance based on static training and testing data collected retrospectively and analyzed *in silico*,^11^ whereas healthcare providers seek to implement MLAs in dynamic, complex real-world clinical settings using live or near-live data.

Theoretical MLA implementation frameworks^12–16^ have attempted to identify key stages, tasks and contextual factors that warrant consideration, but practical translation into end-to-end MLA implementation in clinical practice is uncertain. While systematic reviews have evaluated pre-implementation studies of sepsis MLAs,^6–8^^,^^11^^,^^17^ including interviews generating implementation methods,^18^ none have focused on MLAs actually implemented. Individual studies of deployed MLAs have revealed barriers and enablers that implementation frameworks must incorporate if they are to fully inform successful end-to-end MLA implementation.^18–20^

In this article, we identified and appraised studies of clinically applied sepsis MLAs using systematic methods and then map the serial steps in deployment described in these studies to a recently derived AI implementation framework, called SALIENT (reported in a companion paper^21^ and described in brief below). The mapping sought to clarify where and when barriers, enablers, and key decisions arise within the end-to-end AI implementation process and to validate SALIENT’s capability to guide stakeholders involved in end-to-end MLA implementation.

### Background

**Figure 1.**
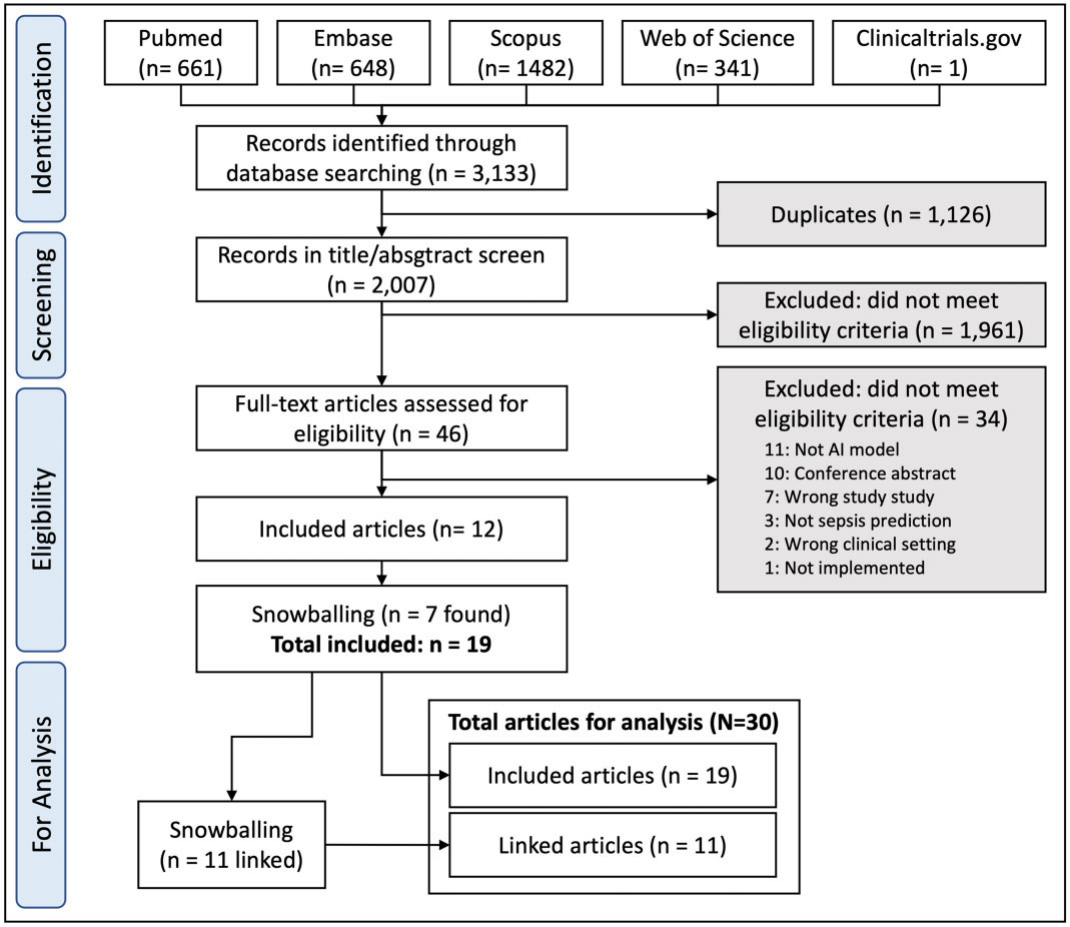
Flowchart for study selection.

The process by which AI interventions are evaluated at any given stage in the implementation cycle is maturing. The recently reported Decide-AI research reporting guidelines depict key stages of algorithm development, evaluation, and implementation,^22^ (Figure 1) which, in the companion paper to this work,^21^ were mapped to Stead et al’s multi-stage approach to translating medical informatics interventions from the lab to the field.^12^ This mapping was used to derive an end-to-end AI implementation framework, called SALIENT (Figure 3 and fully described elsewhere^21^), which accounted for factors found to be missing in many implementation frameworks when subjected to the Stead et al’s taxonomy,^12^^,^^13^^,^^16^^,^^23^^,^^24^ that is, components, both technical and clinical, that need to be developed, evaluated, and integrated over several stages.

The resulting SALIENT stages and associated reporting guidelines are: (I) Definition; (II) Retrospective study—TRIPOD(-AI)^25^^,^^26^; (III) Silent trial—TRIPOD(-AI)^25^^,^^26^; (IV) Pilot trial—Decide-AI^22^; and (V) Large trial/roll-out—CONSORT(-AI).^27^ The SALIENT framework integrates all elements of the reporting standards, and, compared to prior frameworks, renders all components of the end-to-end solution, how and when they integrate, and underlying implementation tasks (not shown here) fully visible. However, similar to most prior frameworks, SALIENT has not been validated in its ability to accommodate reported real-world AI implementation stages, barriers, enablers, and decisions.

## OBJECTIVE

This study had 2 objectives: (1) conduct a systematic review of real-world implementation studies of sepsis MLAs in clinical practice and extract information into how MLA performance, adoption, and different implementation modes were assessed and impacted clinical care processes and patient outcomes; and (2) map the findings regarding barriers, enablers, and key decision points to the different stages and components of the SALIENT AI implementation framework to assess its potential utility for guiding real-world MLA implementation.

## MATERIALS AND METHODS

### Systematic review of sepsis MLA implementation studies

#### Search strategy

The systematic review was performed according to PRISMA guidelines.^28^ Five databases (Pubmed/Medline, EMBASE, Scopus, Web of Science, and clinicaltrials.gov.) were searched between January 1, 2012 and June 23, 2022 for titles and abstracts published in English using keywords and synonyms for: (1) predict; AND (2) sepsis; AND (3) machine learning; AND (4) trial; and NOT (5) child (see Supplementary Appendix SA for complete search queries).

A forwards and backwards citation search (snowballing strategy) was then applied to included papers to identify additional articles that reported new MLAs, or, provided further information about a sepsis MLA described in previously included papers. The latter were labeled *linked* papers, describing MLAs at different stages of implementation, but not considered primary articles.

#### Study selection

Studies of any design were included if: MLAs were applied to adult patients in hospital settings in whom sepsis was formally defined; used live or near-live data; and reported at least one or more algorithm performance metrics (full details in Supplementary Appendix SB). Covidence software^29^ supported a 2-stage screening process with screening of articles by 2 independent reviewers (AHvdV and RJS), with conflicts agreed by 3-way consensus (AHvdV, RJS, and KD); and full-text review by 2 independent reviewers (AHvdV and KD), with selection agreed by 3-way consensus (AHvdV, RJS, and KD). Snowballing was then applied to all included papers and any new or linked papers were identified by AHvdV and verified by KD.

#### Data extraction

Data were extracted independently by 2 authors (AHvdV and KD) using Excel templates, with disagreements resolved by consensus of 2 other authors (RJS and IAS). Extracted data included study metadata, implementation stage, care setting, MLA details including training and validation datasets, performance metrics, outcome definitions and events, and implementation barriers, enablers, and decision points (see Supplementary Appendix SC for more details). Decision points were identified when 2 or more studies chose different options at a certain point in implementation. Barriers were defined as pitfalls or problems hindering implementation success and enablers as tips or activities aiding implementation success. Consensus between authors (AHvdV, PL, and IAS) determined which decisions, enablers and barriers to include as found and which to consolidate under a common title to minimize overlap.

#### Quality assessment

Papers reporting all-cause or sepsis-related mortality underwent risk of bias (RoB) assessment, performed independently by 2 authors (AHvdV and VRK), using either the ROBINS-I tool^30^ for non-randomized studies, or the Cochrane RoB 2 tool^31^ for randomized trials. Mortality was chosen for RoB assessment as it was the most frequently reported and patient-critical measure.

### Application of AI implementation framework

The systematic review findings for barriers, enablers, and decision points were mapped by AHvdV to the stages and elements of the SALIENT implementation framework, followed by a review by IAS and adjustments made where discrepancies were found. An item could be mapped to more than one element and where no obvious element was found to map to, it was recorded.

## RESULTS

### Systematic review of sepsis MLA implementation studies

From 3133 retrieved abstracts, 1126 duplicates were removed, leaving 2007 for screening, from which 12 full-text studies were included for analysis (Figure 1). Most excluded studies were not sepsis prediction studies, or were rule-based rather than AI-based algorithms or were not implemented. An additional 7 articles found by snowballing were selected, yielding a 19 included papers as primary articles, with further snowballing yielding 11 linked papers, giving a total of 30 articles.^32–61^

#### Study characteristics

All 30 studies were published between 2015 and 2022, with 8 algorithm groups (A to H) identified according to the common or named MLA that was the focus of study (Table 1); all were US-based except for Group (C), which was Brazilian. Five groups (A, B, E, F, H) implemented MLAs with a quantitative evaluation (before-after,^33^^,^^50^^,^^59–61^ randomized controlled trial,^58^ 2-armed cohort study,^46^ prospective observational,^33^^,^^44^^,^^48^^,^^53^ retrospective observational^34^). Two other groups (C, D) provided case studies^35^^,^^41^^,^^43^ or qualitative evaluations^42^ and one group (G) reported only post-implementation analyses (retrospective^51^ or difference-in-difference^52^). Groups (B, E) conducted the only multicenter trials with outcomes of more than 10 000 sepsis episodes.^46^^,^^61^ Median trial length was 14 months (range 2–79) and median time between publishing a retrospective study on MLA development and an implementation study was 3 years (range 1–7).

**Table 1.** Listing of studies identified in systematic review, grouped by MLA

Group	Reference	SALIENT stage^a^	Study design (# sites)	Study period	Care location	Outcome^b^ count (prevalence)
**A (EWS 2.0)**	Taylor et al., 2016^32^	II	R (3)		ED	1056 (4.7)
University of Pennsylvania. MLA = random forest; 587 features	Giannini et al., 2019^33^	II	R (3)		Non-ICU	347 (3.3)
		III	PO (2)	Jan–Jun’16	Non-ICU	1540
		V	BA (2)	Jul’16–Feb’17	Non-ICU	2137
	Ginestra et al., 2019^44^	Post	PO (1)	Nov–Dec’16	Non-ICU	NA
**B (Insight)** Dascena Inc and University of California. MLA = various, Inc. Gradient boosted trees^29^ and logistic regression^45^, 6+ variables (laboratory results optional)	Calvert et al., 2016^55^	II	R (1)		ICU	159 (11.4)
	Calvert et al., 2016^56^	II	R (1)		ICU	270 (0.9)
	Desautels et al., 2016^57^	II	R (1)		ICU	2577 (11.3)
	Shimabukuro et al., 2017^58^	IV	RCT (1)	Dec’17–Feb’18	ICU	22 (32.8)
	McCoy et al., 2017^59^	IV	BA (1)	Nov’16–May’17	ALL	921 (NR)
	Calvert et al., 2017^48^	Post	NA		NA	NA
	Mao et al., 2018^36^	II	R (1)		Non-ICU	2142 (2.4)
	Burdick et al., 2018^60^	IV	BA (1)	Jul–Aug’17	ED+ICU	1136 (NR)
	Topiwala et al., 2019^34^	V	RO (1)	Feb–Jun’18	ALL	269 (NR)
	Burdick et al., 2020^61^	V	BA (9)	2017–mid‘18	Non-ICU	14 166 (22.7)
**C (Robot Laura)** Brazil; MLA = NR	Gonçalves et al., 2020^35^	Post	CS (1)	Jan–Jun ‘18	NR	NR
	Scherer et al., 2022^37^	Post	RO (1)	Mar–Sep ‘20	NR	NR
**D (Sepsis Watch**) Duke University. MLA = recurrent neural network; 86 variables	Futoma et al., 2017^38^	II	R (1)		ALL	10 552 (21.4)
	Futoma et al., 2017^39^	II	R (11)		ALL	10 552 (21.4)
	Bedoya et al., 2020^40^	II	R (1)		ALL	813 (18.9)
	Sendak et al., 2020^41^	III/V	CS (3)	Apr’16–Nov’18	ALL	NA
	Sandhu et al., 2020^42^	Post	Q (1)	Jan–Apr’19	NA	NA
	Sendak et al., 2020^43^	Post	CS (1)		NA	NA
**E (TREWScore)** Johns Hopkins University. MLA = Cox proportional hazard; 27 features	Henry et al., 2015^45^	II	R (1)		ICU	2291 (14.1)
	Adams et al., 2022^46^	V	2xAC (5)	Apr’18–Sep’20	Non-ICU	13 680 (2.3)
	Henry et al., 2022^47^	II	R (5)	Jan’16–Mar’18	Non-ICU	3858 (2.2)
		Post	PO (5)	Apr’18–Mar’20	Non-ICU	9805 (2.1)
	Henry et al., 2022^48^	Post	Q (1)	Oct’18–Apr’19	ALL	NA
**F (Sepsis sniffer)** Mayo Clinic. MLA = decision Tree	Harrison et al., 2015^49^	II	R (1)		ICU	86 (29)
	Lipatov et al., 2022^50^	IV	BA (1)	Sep’11–May’18	ED + ICU	1096 (9.8)
**G (ESM)** USA, independent. MLA = Log. Reg.	Wong et al., 2021^51^	Post	R (1)	Dec’18–Oct’19	Unclear	2552 (6.6)
	Schootman et al., 2022^52^	Post	DiD (15)	Jan’16–Jun’19	ALL	6926 (NR)
**H** USA, independent. MLA = naïve Bayes; 5 features	Brown et al., 2016^53^	II	R (2)		ED	549 (0.4)
		Post	PO (1)	Apr’09–Jun’10	ED	352 (0.4)

Study Designs: R: Retrospective; RO: Retrospective Observational; PO: Prospective Observational; BA: Before-after study; 2xAC: Two-Arm Cohort; RCT: randomized control trial; Q: qualitative study; CS: case study; DiD: difference-in-difference analysis.

a Stages in the SALIENT framework: I = problem definition; II = retrospective development; III =Silent trial; IV = Pilot trial; V = Large trial/Roll-out; Post= Post deployment study.

b Outcome definitions are itemized in Supplementary Appendix SF, Table F2.

NA: not applicable; NR: not reported; ED: emergency department; ICU: intensive care unit; ALL: patients from all wards.

Two studies were confined to emergency department settings,^32^^,^^53^ 6 involved only intensive care unit (ICU) patients,^45^^,^^49^^,^^55–58^ and the remainder involved all wards or non-ICU settings. The prevalence of sepsis varied from as low as 2.1%^47^ to as high as 22.7% in non-ICU settings,^61^ and from 11.3%^57^ to 32.8%^58^ in ICU settings.

Most studies reported MLA evaluations at stage II (retrospective),^32^^,^^33^^,^^36^^,^^38–40^^,^^45^^,^^48^^,^^49^^,^^55–57^ 2 at stage III (silent trial),^33^^,^^41^ 4 at stage IV (pilot trial),^50^^,^^58–60^ 3 at stage V (large trial/roll-out),^33^^,^^46^^,^^61^ and 11 reported post-deployment evaluations.^35^^,^^37^^,^^42–44^^,^^47^^,^^48^^,^^51–53^^,^^62^

#### Quality assessment

Eight papers from 5 groups (A, B, E, F, G) were assessed for risk-of-bias (RoB) (see Supplementary Appendix SD). Overall, RoB was serious for 2 groups (A, F), moderate for 2 (E, G) and moderate to critical for one (B). Major sources of bias were potential confounding from additional sepsis control co-interventions, such as staff training in sepsis recognition and management, and other changes in non-sepsis conditions and patient characteristics potentially impacting all-cause mortality at the trial sites. Only 2 trials controlled for these differences in before-after cohorts.^46^^,^^52^

#### Implementation evaluation

Eighty-five distinct metrics were identified across 26 (87%) papers, grouped into 5 evaluation categories: (1) algorithm performance; (2) algorithm adoption; (3) clinical process effects; (4) patient outcome effects; and (5) financial impact. All metrics reported are listed in Supplementary Appendix SE.

##### Algorithm performance and adoption

**Figure 2.**
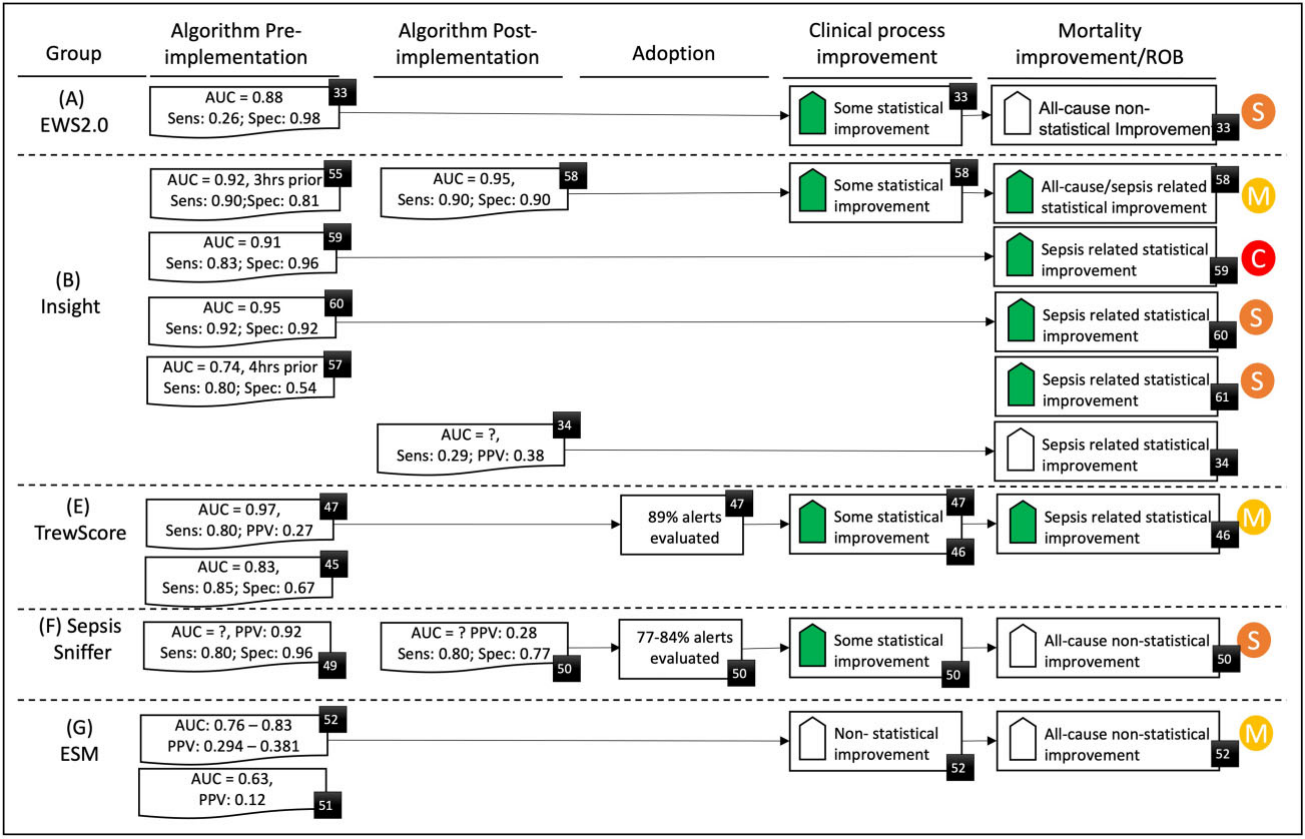
Evaluation results for algorithm performance, adoption, clinical process, and mortality improvement for each MLA group. Figure includes risk of bias (RoB) assessment for studies reporting mortality assessment (M = moderate [some concerns for RoB-2], S = serious, C = critical). Black numbered squares denote the cited paper for that result. AUC: area under the receiver operating curve; Sens: sensitivity; Spec: specificity; PPV/NPV: positive/negative predictive value. Solid shaded up arrow: significant improvement, whereas hollow up arrow: non-significant improvement.

Of 27 performance metrics, sensitivity and positive predictive value were most commonly reported (7 groups, 18 and 9 papers, respectively), closely followed by area under the receiver operating curve (AUROC) and specificity (6 groups, 17 and 13 papers). Most (66%) post-implementation studies did not report real-world MLA performance (Figure 2), and of the 3 that did, one reported improved MLA performance^58^ while the other 2 showed marked declines,^34^^,^^50^ and similarly for the external validation study of the EPIC tool (Group G).^51^

MLA adoption, measured as the proportion of alerts clinicians responded to, was only reported in Group (E) at 89%, Group (F) at 77%–84% and Group (C) at 100%.

##### Clinical impact

Of 36 distinct clinical process outcomes reported across 9 papers in 5 groups, the most common were median lead time to first antibiotic use (5 papers, 4 groups), the 3-h sepsis care bundle compliance rate and the increases in antibiotic use (both 3 papers, 3 groups).

Ten different patient outcomes were reported, most commonly mortality and length of hospital stay (LOS) (both 5 groups, 9 papers). All 9 papers^34^^,^^44^^,^^46^^,^^50^^,^^52^^,^^58–61^ reported decreased mortality, be that all-cause, sepsis-related or both, although this was statistically significant only for 5 studies: Group (B), both all-cause^58^ and sepsis-related,^59–61^ and Group (E), for sepsis-related only,^46^ both involving a large samples of >13 500 septic patients. Only Group (E) adjusted their findings for differences between cohorts in patient characteristics. Only Group (B) performed more than one independent post-implementation mortality study, with all 5 studies showing improved mortality,^58–61^ although Topiwala et al^34^ reported poor post-implementation MLA performance and no significant improvement in mortality.

Of the 2 groups reporting significantly improved sepsis-related mortality, only Group (E) reported strong MLA adoption (89%) and significant decreases in antibiotic lead time.^46^ Group (B) reported no adoption data and only their smallest study reported improvement in a single process outcome: lead time to antibiotic use.^58^ Despite group (F) reporting high adoption rates (77% to 84%) and significantly improved rates of sepsis care bundle compliance, post-implementation the MLA specificity dropped markedly, from 96% to 80%, and there was no significant change in all-cause mortality.^50^

### Identification of implementation barriers, enablers, and decision points and mapping to SALIENT AI implementation framework

Barriers, enablers, and decision points provide real-world evidence of factors that are reported by practitioners and can impact MLA implementation success.

#### Barriers and enablers

We identified 14 unique barriers (Table 2) and 26 unique enablers (Table 3) from a total of 70 mentions across all studies. The most common barriers, identified by at least 3 groups, were lack of clinician trust (B1), alert fatigue (B4) and dismissal of alerts, mainly because clinicians perceived no clinical signs of deterioration (B3). However, 8 barriers were unique to a single group (D), and despite more enablers than barriers, just 2 groups (D, E) provided 80% of the group-level enabler instances.^41–43^^,^^48^ The most commonly reported enabler was frequent communications to raise awareness of the MLA during and after clinical trials (E4), with clinician involvement (E1), improvement cycles (E3), clinical champions (E5), and test versions for training (E6) reported by more than 2 groups. Overall, 90% of all barriers and enablers were AI task agnostic, with just one barrier (B12) and 3 enablers (E2, E11, E26) specific to sepsis prediction.

**Table 2.** Implementation barriers

	Barriers (stage)	Component or element	A	B	C	D	E	F	G	H	Total
B1	Lack of clinician trust (IV+)^18^	ICA	2		1	1	1				5/4
B2	MLA retraining concerns: Feedback loops arise when alerts lead to timely treatment. (IV+)	AI				1					1/1
B3	Alerts dismissed for wrong reasons, for example, patients with no sepsis symptoms or with higher acute complexity (IV+)	CW	2				2			1	5/3
B4	Alert fatigue (II+)^18^	AI; CW; ICA	1			1		1			3/3
B5	Differential nurse/Doctor role, perceptions of role and value (IV+)^18^	CW; ICA	1			1					2/2
B6	Inherent limitations of EHR data, which can be plagued by missingness, inaccuracies, and changes in practice patterns over time (II+)	DP	1			1					2/2
B7	Data entry delays, leading to delayed predictions (III+)	DP			1						1/1
B8	Inventors/company equity owners may have COI and inadvertently act in bias ways towards the evaluation of their system (III+)	ET				1					1/1
B9	Surveillance bias: important to monitor impact of Alerts on non-septic patients for over-prescription of antibiotics (IV+)	ET; EM					1				1/1
B10	Substantial cost involved for infrastructure, implementation personnel time and ongoing maintenance (All)^18^	ICA; GOV				1					1/1
B11	Lack of individual proficiency of health professionals in the use of hardware and software (IV+)	CW; ICA			1						1/1
B12	Clinicians perceive they are better at diagnosing sepsis than the AI and the alert occurs after they already suspect (IV+)^18^	CW; AI; ICA	1			1					2/2
B13	Lack of machine learning foundational knowledge and firsthand experience (II+)^63^	CW; ICA				1					1/1
B14	Clinician concern over reliance on system (IV+)^63^	GOV; QS; ICA					1				1/1
	**Total papers**		8	0	3	9	5	1	0	1	27
	**Count of barriers identified by the group**		6	0	3	9	4	1	0	1	24

For reach barrier, the number of papers that identify the barrier within each group are noted in columns A to H. The totals column is in the format of: total number of papers/total number of groups. The associated element or component in the derived framework is also identified where ICA: Implementation, change management & adoption; AI: AI model; CW: clinical workflow; DP: data pipeline; GOV: governance; QS: Quality & safety; EM: Evaluation and monitoring. Beside each barrier is listed the stage in parentheses, that is associated with that barrier.

**Table 3.** Implementation enablers: for reach enabler, the number of papers that identify the enabler within each group are noted in columns A to H

	Enablers (stages)	Compo-nent	A	B	C	D	E	F	G	H	Tot.
E1	Clinician involvement is essential at all stages of model/HCI development and integration into clinical workflow (II+)^18^^,^^63^	AI; CW, HCI; GOV				2	2				4/2
E2	Better AI model training methods: for negative cases, use portion of patient journey when sick, not within 6h or discharge (II/III)	AI				1					1/1
E3	Conduct improvement initiatives (PDSA) cycles during implementation to quickly garner and act on clinical feedback (III+)	CW; QS; ICA								1	3/2
E4	Frequent communications to increase awareness during and after trial, for example, weekly meetings, emails, educational sessions giving progress and setting next goals and highlighting urgent need. (IV+)^18^	ICA		1	1	1	2				5/4
E5	Appoint clinical champions to advocate for the tool (II+)	ICA				1	2				3/2
E6	Create a test version of the application to train clinicians and multi-channel training approaches incl. web (III)	CW				1	1				2/2
E7	Use a tablet with training loaded, plus feedback mechanism so training could occur on-the-job (IV+)	CW				1					1/1
E8	Implement alternative workflows during peak hours (IV+)^18^	CW					1				1/1
E9	Clinicians were taught how to interpret risk scores (IV+)	CW; ET		1							1/1
E10	Iterative approach to design of clinical workflow, human-computer interface and AI model (II+)	AI; CW; HCI				1					1/1
E11	Visually delineating sepsis risk into colors (red cards as high risk, etc) and tracking patients across distinct tabs (patients to be triaged, screened out for sepsis, and those in the sepsis bundle) (III)	HCI				1					1/1
E12	Perform post-implementation interview (study) to identify improvements (IV+)	CW; HCI; QS; ICA				1					1/1
E13	HCI was augmented by completion and fallout indicators to visually guide the clinician to timely and appropriate care (III)	CW, HCI						1			1/1
E14	Report the number of cases the AI detects that clinicians miss: clinicians requested this—build trust (IV+)	EM; ICA				1	2				3/2
E15	Track and monitor data and model drift (III+)	QS; EM				1					1/1
E16	Work with regulatory officials to ensure the solution is qualified as CDS and not a diagnostic medical device (I+)	RL				2					2/1
E17	Establish a multi-disciplinary governance committee to promote usage, track compliance, provide training and plan for post-trial sustainability; and an external data safety board to oversee safety and AI efficacy (I+)	GOV				1					1/1
E18	A dedicated full-time role can work with frontline clinicians and stakeholders to integrate the tool (IV+)	ICA; CW			1	1					2/2
E19	Strong support from senior leadership (I+)^18^	ICA; GOV				1					1/1
E20	Establish a transdisciplinary team of data scientists, statisticians, hospitalists, intensivists, ED clinicians, RRT nurses, and information technology leaders and develop capabilities across domains (I+)	ICA; GOV				1					1/1
E21	Staggered deployment across sites (V+)	ICA					1				1/1
E22	Although numbers and statistical trends were used as evidence, individual patient cases were important to frontline clinicians (IV+)	ICA				1					1/1
E23	Carefully navigate the lines of professional authority that physicians have toward the care of patients. Tool described as supporting physicians and nurses. The term AI was never used (I+)	ICA; ET				2					2/1
E24	Trust in the model increased as the clinician experienced the algorithm make correct predictions (IV+)	ICA				1					1/1
E25	A “Model Facts” sheet designed to convey relevant information about the model to clinical end users; (II)	ET; AI; ICA				1					1/1
E26	System sepsis monitoring was experienced as alleviating demands on attention and cognition (IV+)	ICA					1				1/1
	**Total papers**		0	4	2	23	12	1	0	1	43
	**Count of group enablers**		0	3	2	20	8	1	0	1	35

The totals column is in the format of: total number of papers/total number of groups. The associated components in the SALIENT framework are also identified where ICA: Implementation, change management and adoption; AI: AI model, HCI: human–computer interface, CW: clinical workflow, DP: data pipeline, GOV: governance; ET: Ethics; EM: evaluation and monitoring; RL: Regulatory& legal and QS: Quality & safety. Beside each enabler is listed the stage in parentheses, that is associated with that enabler.

**Figure 3.**
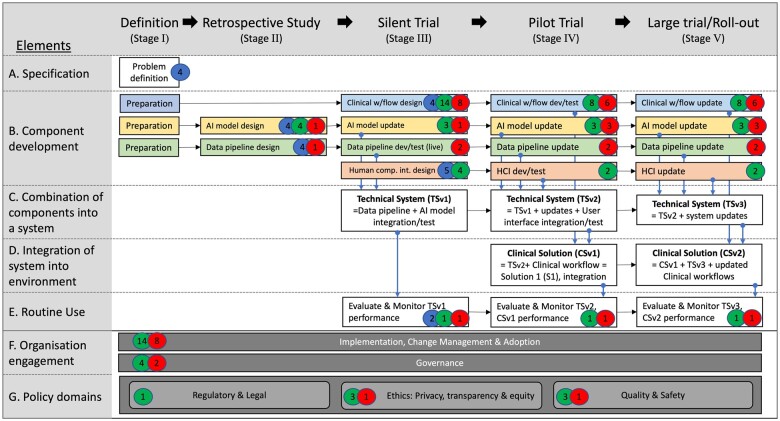
The number of barriers (red badge), enablers (green badge), and decision points (blue badge), denoted by the number within the badge, mapped to each stage and component of the SALIENT AI implementation framework. Implementation stages are labeled in the title row from left to right. The color-coded solution components are developed in row B, and consist of Clinical workflow (blue), AI model (yellow), data pipeline (green), and human-computer interface (red). The components are integrated in rows C and D, and rows F and G describe cross-stage elements required throughout the entire implementation process, such as governance and quality and safety assurance. w/flow: workflow; dev/test: development & test; HCI: human computer interface.

All barriers and enablers could be mapped to the SALIENT AI implantation framework (see Figure 3). All barriers (*n* = 14) were located between the silent trial stage (III) and the large trial or roll-out stage (V). Most enablers and barriers were identified for the clinical workflow solution component (*n* = 6 and *n* = 8, respectively in stages IV and V) and the cross-stage element, ‘Implementation, change management and adoption’ (*n* = 8 and *n* = 14, respectively). No barriers were identified that related to the regulatory and legal policy domain or the human computer interface solution component, whereas enablers were identified in all solution components and all cross-stage policy and organizational elements.

#### Decision points

Twenty-two decision points were identified in our review, with 17 identified by at least 2 groups; all were mapped to the SALIENT implementation framework (Table 4) and depicted in Figure 3.

**Table 4.** Decision points identified for each component in the SALIENT framework

	Decision Points	A	B	C	D	E	F	G	H	Tot
**Definition**										
D1	Which patients? Age; location: ICU, ED, all non-ICU	Identified by differences across papers								
D2	What to predict? sepsis, severe, shock? Should you prioritize on mortality? Patients admitted with sepsis and/or hospital acquired sepsis?				1	1	1			3/3
D3	What objective/bundle compliance, early identification, mortality/LOS—primary and secondary outcomes; anti-microbial mis-use (flow on to model)	1			1				1	3/3
D4	What is the minimum expected performance for alarms? precision v sensitivity?		1			1		1		3/3
**AI model**										
D5	Which model: ML vs DL (explainable, earliness of prediction) and where trained	1			2	1				4/3
D6	Which features: simple vs complex, set-in-stone or changeable. Noting this will impact earliest first prediction: immediately at ED or later?	1	3						1	5/3
D7	How early to target alerts? (too early—no symptoms/signs, too late, no clinical utility)	2			1	2	1		1	7/5
D8	What outcome basis for Train/Evaluate?	1			3		1			5/3
**Data pipeline**										
D9	What data access approach to use: direct or separate				2					2/1
D10	Whether inhouse vs external platform/product/solution				2					2/1
D11	What methods of data imputation to use				2					2/1
D12	What level of pipeline sophistication can be supported: model performance vs engineering effort				1					1/1
**Clinical workflow**										
D13	Whether dedicated vs distributed model of alert handling		1		2	1				4/3
D14	What determines the setpoint decision		1			1				2/2
D15	How to deal with ambiguity over alerted patients that have NOT decompensated	2				2			1	5/3
**Human–computer interface**										
D16	Whether integrated with EMR or not and if not—are tablets/phones allowed				3	1				4/2
D17	Whether individual notification (hard alert) or aggregated dashboard (soft alert)				2	2	1			5/3
D18	Which alert timing: suppression of alerts after first alert; one-time or repeat	1	2							3/2
D19	Whether to provide clinician feedback or not									
D20	Whether prediction is explained or not	2	1		3	2				8/4
**Evaluation and monitoring**										
D21	Which metrics to use	Identified by differences across papers								
D22	What process to follow: Silent trial or not and which trial method				1	2		1		4/3
	**Count of papers**	11	9	0	26	16	4	2	4	72
	**Count of group decisions**	8	6	0	14	11	4	2	4	49

The numerals refer to the number of papers by group (A -> H) that discuss a particular decision. The totals column is in the format of: total number of papers/total number of groups.

EHR: electronic health record; ML: machine learning; DL: deep learning; ICU: intensive care unit; ED: emergency department.

##### Definition decision points (D1-D4)

The target population and care locations were reported by 7 groups (D1); all included the ED, 5 added the ICU or general wards and 4 targeted all areas. No study reported use of different algorithms for the ICU and non-ICU wards, despite ICUs collecting more data elements at higher frequency. Other decisions related to identifying all hospitalized patients with sepsis, including at ED presentation, or only those acquiring it whilst in hospital,^38^ and whether to identify only patients at higher risk of mortality for prioritized clinical review, thus minimizing clinician workload.^46^

Twenty-six different definitions of sepsis were used (see Supplementary Appendix SF, Table F2), ranging from sepsis to severe sepsis to septic shock (D2). The prime purpose for implementing sepsis MLAs varied which in turn determined how they were trained and evaluated (D3),^43^ with evaluation metrics and success criteria varying depending on whether increasing sepsis care bundle compliance,^50^ providing a sepsis detection and management system,^41^ reducing anti-microbial overuse^52^ or decreasing patient mortality and LOS were primary objectives.^34^^,^^46^^,^^58^^,^^59^^,^^61^ The algorithm objective also determined the minimum expected performance for the MLA (D4), in terms of sensitivity (proportion of septic cases detected) and false alarms (proportion of non-septic cases misidentified as sepsis). Different thresholds were chosen according to the anticipated impacts on clinical processes and clinician workload and adoption.^60^^,^^61^

##### AI model decision points (D5–D8)

We identified 1 statistical AI model (E), 5 ML models (A, B, F, G, H), 1 deep learning (DL) model (E), and 1 unknown (C), with no single model being utilized by more than one group. Selection of model type (D5) varied according to perceived accuracy and adoption based on the level of model explainability,^41^^,^^45^^,^^55^ but with trade-offs according to the model’s ability to support time-series data,^38^^,^^40^ accommodate large, high-dimensional datasets,^48^ and demonstrate better performance.^41^ Group (D) reported clinicians were willing to sacrifice explainability for more accurate predictions and better standardized treatment of all sepsis cases,^41^ while Ginestra et al found clinicians most wanted transparency regarding the predictive features generating the alerts.^44^

Deciding which features to input and how simple (eg, vital signs only) or complex (eg, waveform data and laboratory results) they are was seen to influence model generalizability (D6) to different care locations.^38^^,^^57^^,^^58^^,^^62^ Group (B) supported different variables for different sites, claiming flexibility,^62^ although new models needed to be trained, validated and maintained at each site. Another decision was how quickly the MLA needed to make its first prediction after admission (D7), contingent upon the availability of the required data, with potential delays, for example, in obtaining laboratory investigation results.^53^

Predicting onset of sepsis as early as possible involved trade-offs between: (1) alerts that were too early, where clinicians may not have known what to do, and therefore dismissed the alerts^33^^,^^41^^,^^42^^,^^44^^,^^47^^,^^50^^,^^53^; and (2) alerts that were too late for patients for whom clinicians already suspected sepsis and had initiated appropriate care bundles (in one study up to half^44^), thereby diminishing its clinical utility.^44^^,^^50^ The choice also had implications for MLA training and evaluation (D8).

##### Data pipeline decision points (D9-D12)

Only group (D) contributed to data pipeline decisions for which only 2 barriers (B6, B7) and no enablers were reported. Decisions had to be made (D9) about how to access the data: direct from the Electronic Health Record (EHR), which could entail partnering with the vendor, or indirectly from a real-time data warehouse or various feeder systems.^43^ Similarly, whether to develop and implement the MLA in-house or use an external vendor (D10), which involved weighing up the capability to future-proof the organization for future AI solutions^41^ versus implementation and maintenance challenges arising from separate ownership of the input data and the AI model.^41^ The required level of data pipeline sophistication, including data imputation (D11) and transformation, also necessitated trade-offs between engineering effort versus model performance (D12),^41^ with group (D) having to remove a data imputation pipeline because of its complexity.^40^

##### Clinical workflow decisions (D13-D15)

Whether alerts were to be sent to and managed by dedicated clinical staff (centralized approach) or sent directly to clinicians responsible for individual patient care (distributed approach) varied across studies (D13). Five (A, C, E, F, G) groups chose the former, whereas Sandhu et al found physicians preferred the latter, having a nurse contact them directly, often in-person, rather than by means of EHR-generated alerts which imposed greater cognitive load and interruptions.^42^ However, the same physicians still saw nurse contacts as disruptive, while nurses found physicians often too busy to contact.^42^ Having a dedicated clinician receive calls minimized alarm fatigue,^41^ but group (E) found a distributed approach more scalable for monitoring multiple conditions, more feasible in small-staffed sites, and more able to provoke bedside reviews,^48^ although clinicians often regarded the numbers of reviews as unmanageable.^59^

The MLA alert threshold or setpoint determining the numbers of alerts was a key decision impacting clinician workload (D14). Group (E) utilized an improvement cycle to decide on the alert threshold at each local implementation site in improving clinician adoption.^47^^,^^59^

Related to the timing of alerts (D7), decisions about what actions clinicians should take for alerts involving patients showing no symptoms or signs of sepsis proved problematic (D15), as unclear roles and responsibilities constituted potential barriers to adoption (B3, B5).^33^^,^^44^

##### Human computer interface (HCI) decisions (D16–D20)

How algorithm predictions were presented to clinicians and whether they were accompanied by additional information or even recommendations varied between groups. The HCI options comprised: (1) an alert only (Groups B, H), or with optional attached information (Group A) sent directly to clinicians via messaging systems (phones, e-mails, personal tablets) (D16); (2) content integrated within existing EHRs (Groups E, G); or (3) an external dashboard or application (Groups C, D, F) (D17). Integration into an EHR relied on organisations having a single EHR, otherwise multiple HCIs were required. Also, many EHRs did not have in-built capacity to support complex MLAs,^41^ whereas external dashboards conferred flexibility to design a bespoke solution that could also support mobile devices,^41^ although requiring clinicians to switch between applications interrupting workflows.^48^ The type of alert (D17) varied between hard alerts (such as a pop-up directive) requiring clinicians to immediately respond, and soft alerts (such as colored icons) that were more easily managed.^41^^,^^42^^,^^46^^,^^48^ No group indicated which method prompted more appropriate clinical actions and conferred better clinical outcomes.^50^

Whether alerts were allowed to fire once or repeatedly until deactivated (D18) also varied between groups. The EWS2.0 (Group A) used a one-time alert, but found clinician evaluation of patients often occurred some hours after the alert fired.^33^ Group (F) implemented completion and fall-out indicators for single alerts to visually guide clinicians to more timely review.^50^ Group (B) supported multiple alerts for the same patient, but incorporated a snooze feature to suppress alerts within 6 h of the first alert.^59^^,^^61^ Whether to include more information about what caused alerts, versus just firing alerts alone (D20) had implications as to how the algorithm was trained. The decision by groups (E, F) to enable clinicians to feedback whether they thought the alert represented sepsis or something else (D19) enabled implementation teams to evaluate clinical utility, and provide feedback to clinicians about missed sepsis cases, which incentivized greater adoption.^42^^,^^48^

##### Evaluation decisions (D21, D22)

Evaluation decisions (D21) proved challenging as most groups omitted pre- and post-implementation evaluations of MLA performance using the same metrics. If done, it would have enabled linking of MLA performance with changes in clinical care or outcomes (Figure 2). Pre-implementation studies reported AUROC ranging from 0.63^51^ to 0.97^47^ but only one post-implementation group (B) study^58^ reported AUROC of 0.95, which was similar to pre-implementation studies.^55^^,^^59^^,^^60^

In regard to pre-deployment silent or shadow trials evaluating algorithm performance against conventional clinical judgment in a live-data environment (D22),^33^ 3 groups (A, D, E) conducted such trials for 6, 3 and an unknown number of months, respectively, during which algorithm validation was undertaken as well as end-to-end testing of the model, the data pipeline, the HCI and the clinical workflow.^41^^,^^48^

## DISCUSSION

### Systematic review of sepsis MLA implementation studies

The systematic review served to learn how MLA performance, adoption, and different implementation modes were measured and how they impacted clinical care processes and patient outcomes. We found MLAs have potential to reduce mortality, but no definitive causal relationship has been demonstrated. At a minimum, the causal chain requires a high performing (high sensitivity/low false alarm) implemented MLA, clinician adoption and resulting positive changes to clinical processes (see Figure 2). Two groups (B, E) could demonstrate at least 2 of these, together with a significant reduction in mortality but only Group E reported definitive evidence of MLA adoption.

Demonstrating a causal link was limited by: (1) Non-randomized study designs being subject to confounding bias, such as sepsis awareness programs accompanying MLA implementation; and (2) Infrequently reported and non-standardized MLA performance metrics post-implementation, which, when they were reported, often showed decreased accuracy. Given these limitations, it remains unclear whether MLAs were responsible or needed for improved mortality. In a meta-review of 55 observational studies of sepsis reduction programs using guideline-based care bundles,^64^ a significant 34% overall reduction in mortality was achieved despite the absence of digitally embedded sepsis screening or alert tools in most studies (43/55, 78%).

Other important study findings were, firstly, clinical process improvements after MLA implementation did not always result in better patient outcomes, likely due to different clinical process improvement metrics (*N* = 36). However, significant reductions in just one metric, median lead time from alert to first antibiotic, did coincide with significant reductions in mortality,^46^^,^^47^^,^^58^ suggesting this as an important indicator of MLA implementation success.

Second, it remains unclear whether MLA model choice impacts implementation success. Seven different algorithms were implemented with 5 reporting improved clinical indicators and mortality outcomes. The level of MLA performance post-implementation appears to be more important than choice of algorithm in predicting effectiveness. Across 2 different MLAs (Groups B and F), only the algorithm with high post-implementation performance was associated with significant mortality improvement.^50^^,^^58^ Similar results were seen for 2 independent implementations of the same algorithm (Group B).^34^^,^^58^

Third, the choice of outcome definition, in this case sepsis, is critical as it can directly influence algorithm performance measures, particularly specificity.^6^ Definitions of sepsis varied from initial systemic inflammatory process (eg, Sepsis-1 definition)^3^ to multi-organ dysfunction (eg, Sepsis-3 definition)^65^ reflecting a later, more advanced state of the illness. Importantly, the concern here is diagnosing sepsis (ie, using a diagnostic predictive algorithm) rather than predicting the likelihood of sepsis occurring in a patient before the inflammatory process begins (ie, a prognostic predictive algorithm).^66^

Fourth, how algorithm predictions are presented to clinicians, and the extent to which they are accompanied by additional information or even recommendations are key determinants of clinician acceptance.^67^

### Mapping to SALIENT AI implementation framework

The second study objective was to map the review findings to the SALIENT framework to validate its coverage of important real-world implementation factors. Unlike in similar reviews,^6–8^^,^^11^^,^^17^^,^^68^^,^^69^ we conducted a novel 2-stage review wherein the second stage we identified related studies before or after the principal deployment study, which provided studies across the end-to-end MLA implementation process. We found the findings of each study could be mapped to one or more stages within SALIENT and that all SALIENT stages were utilized across all studies, indicating that SALIENT’s implementation stages are both necessary and sufficient for real-world sepsis MLA implementation.

Secondly, every barrier, enabler, and decision identified in the review could be located to a stage (I-V) and either components (AI model, data pipeline, clinical workflow, HCI) or elements (A–G) within SALIENT. Knowing in advance what decisions are required (for example as a checklist), when they need to be made and in relation to which part of the implementation process is novel and could be informative to those engaged in AI implementation planning. We also found that most of the decision points, barriers and enablers identified were not specific to sepsis prediction, but were AI-task agnostic, suggesting SALIENT may have application for non-sepsis MLA implementation projects.

### Strengths and limitations

As far as we know, our study is the first attempt to undertake a systematic review of sepsis prediction algorithms deployed in clinical settings, to identify barriers, enablers, and key decision points, and to map these to a single, inclusive, end-to-end implementation framework. The resulting framework and mapped items render these key decisions and contextual factors explicit, ordered and transparent, address gaps in current implementation guidance and offers a pragmatic staged approach for use by clinicians, informatics personnel and managers. Limitations relate to the small number of empirical studies of deployed sepsis-prediction algorithms, under-reporting of post-implementation performance metrics, focus on adult hospital settings, and potential publication bias from under-reporting of other sepsis MLA implementation studies.^18^ Although risk of bias for mortality reporting studies was moderate to high, all studies, including the 3 lowest bias papers,^46^^,^^52^^,^^58^ reported numerical reductions in mortality, with 5 being significant.^46^^,^^58–61^

## CONCLUSIONS

Our systematic review indicates that implementing MLAs within adult hospital care settings to predict sepsis has potential to reduce mortality, but no definitive causal link has been demonstrated. Implemented MLAs were few and only 2 provided some evidence of causation. The types of MLA models employed mattered less than their implementation accuracies and ability to alert clinicians to order antibiotics earlier.

This study also validated the SALIENT framework demonstrating real-world MLA implementation barriers, enablers, and decisions could be mapped to its constituent stages and components. Our findings highlight that AI implementation success has many more dimensions than the types of MLA employed, including evaluation methods and stages and the many decisions required throughout the multi-stage process. SALIENT may provide a roadmap for stakeholders to identify these stages, components and decisions which, with more robust studies, may be shown to conclusively link MLA implementation with significant improvement in patient outcomes. The SALIENT framework also has potential application to other MLA algorithms seeking to identify patients at risk of other acute hospital acquired conditions.

## Supplementary Material

ocad075_Supplementary_Data

## Data Availability

There are no new data associated with this article.
