# Supplementary material for: Deployment of machine learning algorithms to predict sepsis: systematic review and application of the SALIENT clinical AI implementation framework
Source: J Am Med Inform Assoc. 2023 May 12;30(7):1349–61. doi: 10.1093/jamia/ocad075 (PMC10280361; doi:10.1093/jamia/ocad075)
Supplement: ocad075_Supplementary_Data [file ocad075_supplementary_data.docx]

Supplementary Appendices

Table of Contents

[Appendix A: Search details 2](#_Toc117064281)

[Pubmed search details 2](#_Toc117064282)

[Embase search details 2](#_Toc117064283)

[Scopus search details 3](#_Toc117064284)

[Web of Science search details 4](#_Toc117064285)

[Appendix B. Selection criteria 6](#_Toc117064286)

[Appendix C. Extracted data items 7](#_Toc117064287)

[Paper Information 7](#_Toc117064288)

[Retrospective dataset details 7](#_Toc117064289)

[Validation dataset details 7](#_Toc117064290)

[Implementation details 7](#_Toc117064291)

[Appendix D Risk of Bias Assessment 9](#_Toc117064293)

[Appendix E: Extracted Evaluation Metrics 11](#_Toc117064294)

[Appendix F: Sepsis definitions employed by each included and linked study 14](#_Toc117064298)

[Table F1: Criteria utilised by papers, together with references, if provided 14](#_Toc117064299)

[Table F2: Sepsis definitions used by each paper 16](#_Toc117064300)

# Appendix A: Search details

## Pubmed search details

| **Search number** | **Query** | **Results** |
| --- | --- | --- |
| 6 | #1 AND #2 AND #3 AND #4 NOT #5 AND (("2012/01/01"[Date - Publication] : "2023/01/01"[Date - Publication])) | 661 |
|  |  |  |
| 5 | (("Adolescent"[Mesh] OR "Child"[Mesh] OR "Infant"[Mesh] OR adolescen*[tiab] OR child*[tiab] OR schoolchild*[tiab] OR infant*[tiab] OR girl*[tiab] OR boy*[tiab] OR teen[tiab] OR teens[tiab] OR teenager*[tiab] OR youth*[tiab] OR pediatr*[tiab] OR paediatr*[tiab] OR puber*[tiab]) NOT ("Adult"[Mesh] OR adult*[tiab] OR man[tiab] OR men[tiab] OR woman[tiab] OR women[tiab])) NOT (animals[mh] NOT humans[mh]) | 2,261,455 |
|  |  |  |
| 4 | "clinical trials as topic"[MeSH Terms] OR trial*[tiab] OR "clinical study"[Publication Type] OR "clinical studies as topic"[MeSH Terms] OR clinical study[tiab] OR "prospective studies"[MeSH Terms] OR prospective stud*[tiab] OR "pilot projects"[MeSH Terms] OR pilot stud*[tiab] OR "controlled before-after studies"[MeSH Terms] OR controlled before-after stud*[tiab] OR trial[tiab] OR roll-out[tiab] OR random controlled trial*[tiab] OR RCT[tiab] OR controlled trial[tiab] OR non-random* trial[tiab] OR prospective[tiab] OR prospective validation[tiab] OR implementation[tiab] OR pre-post implementation[tiab] | 3,076,928 |
|  |  |  |
| 3 | "Diagnosis, Computer-Assisted"[Mesh] OR "Early Diagnosis"[Mesh:NoExp] OR predict*[tiab] OR detect*[tiab] OR identif*[tiab] OR early diagnos*[tiab] | 7,197,500 |
|  |  |  |
| 2 | "Algorithms"[Mesh] OR "Artificial Intelligence"[Mesh] OR "Decision trees"[MeSH] OR "Decision Support Systems, Clinical"[Mesh] OR "Decision Support Techniques"[Mesh] OR "Decision Support Systems, Clinical"[Mesh] OR machine learning[tiab] OR prediction model*[tiab] OR neural network*[tiab] OR deep learning[tiab] OR artificial intelligence[tiab] OR AI[tiab] OR decision tree*[tiab] OR computational intelligence[tiab] OR machine intelligence[tiab] OR algorithm*[tiab] OR big data[tiab] OR bayesian[tiab] OR naïve bayes[tiab] OR k-nearest neighbour[tiab] OR decision support[tiab] OR random forest[tiab] OR support vector machine[tiab] OR SVM[tiab] OR Xgboost[tiab] OR adaboost[tiab] OR gradient boosting machine*[tiab] OR regression tree*[tiab] OR least squares[tiab] OR stepwise regression[tiab] OR LSTM[tiab] or RNN[tiab] | 881,173 |
|  |  |  |
| 1 | "Systemic Inflammatory Response Syndrome"[Mesh] OR "Sepsis"[Mesh] OR "Shock, Septic"[Mesh] OR sepsis[tiab] OR septic[tiab] OR systemic inflammatory response syndrome[tiab] OR SIRS[tiab] OR septicemia*[tiab] OR SOFA[tiab] OR sequential organ failure assessment[tiab] OR qSOFA[tiab] | 248,524 |

## Embase search details

| **No.** | **Query** | **Results** |
| --- | --- | --- |
| #10 | #1 AND #3 AND #4 AND #5 NOT #8 | 648 |
|  |  |  |
| #9 | #1 AND #3 AND #4 AND #5 | 772 |
|  |  |  |
| #8 | (adolescen*:ti,ab,kw OR child*:ti,ab,kw OR schoolchild*:ti,ab,kw OR infant*:ti,ab,kw OR girl*:ti,ab,kw OR boy*:ti,ab,kw OR teen*:ti,ab,kw OR youth*:ti,ab,kw OR pediatr*:ti,ab,kw OR paediatr*:ti,ab,kw OR puber*:ti,ab,kw) NOT (adult*:ti,ab,kw OR man:ti,ab,kw OR men:ti,ab,kw OR woman:ti,ab,kw OR women:ti,ab,kw) | 2438896 |
|  |  |  |
| #7 | #1 AND #2 AND #3 AND #4 AND #5 | 229 |
|  |  |  |
| #5 | trial*:ti,ab,kw OR 'clinical study':ti,ab,kw OR 'prospective stud*':ti,ab,kw OR 'pilot stud*':ti,ab,kw OR 'controlled before-after stud*':ti,ab,kw OR 'roll-out':ti,ab,kw OR 'random controlled trial*':ti,ab,kw OR rct:ti,ab,kw OR 'controlled trial':ti,ab,kw OR 'non-random* trial':ti,ab,kw OR prospective:ti,ab,kw OR 'prospective validation':ti,ab,kw OR implementation:ti,ab,kw OR 'pre-post implementation':ti,ab,kw | 3096251 |
|  |  |  |
| #4 | predict*:ti,ab,kw OR detect*:ti,ab,kw OR identif*:ti,ab,kw OR 'early diagnos*':ti,ab,kw | 9299314 |
| #3 | 'machine learning':ti,ab,kw OR 'prediction model*':ti,ab,kw OR 'neural network*':ti,ab,kw OR 'deep learning':ti,ab,kw OR 'artificial intelligence':ti,ab,kw OR ai:ti,ab,kw OR 'decision tree*':ti,ab,kw OR 'computational intelligence':ti,ab,kw OR 'machine intelligence':ti,ab,kw OR 'algorithm*':ti,ab,kw OR 'big data':ti,ab,kw OR bayesian:ti,ab,kw OR 'naïve bayes':ti,ab,kw OR 'k-nearest neighbour':ti,ab,kw OR 'decision support':ti,ab,kw OR 'random forest':ti,ab,kw OR 'support vector machine':ti,ab,kw OR svm:ti,ab,kw OR xgboost:ti,ab,kw OR adaboost:ti,ab,kw OR 'gradient boosting machine*':ti,ab,kw OR 'regression tree*':ti,ab,kw OR 'least squares':ti,ab,kw OR 'stepwise regression':ti,ab,kw OR lstm:ti,ab,kw OR rnn:ti,ab,kw | 773856 |
|  |  |  |
| #1 | (sepsis:ti,ab,kw OR septic:ti,ab,kw OR sirs:ti,ab,kw OR 'systemic inflammatory response syndrome':ti,ab,kw OR septicemia:ti,ab,kw OR 'sequential organ failure assessment':ti,ab,kw OR sofa:ti,ab,kw OR qsofa:ti,ab,kw) AND [2012-2023]/py | 147508 |

## Scopus search details

#1: 127,810 AT 14:31 ON 23 JUNE 2022

TITLE-ABS-KEY ( sepsis ) OR TITLE-ABS-KEY ( septic ) OR TITLE-ABS-KEY ( sirs ) OR TITLE-ABS-KEY ( "systemic inflammatory response syndrome" ) OR TITLE-ABS-KEY ( septicemia ) OR TITLE-ABS-KEY ( "sequential organ failure assessment" ) OR TITLE-ABS-KEY ( sofa ) OR TITLE-ABS-KEY ( qsofa ) AND ( LIMIT-TO ( PUBYEAR , 2024 ) OR LIMIT-TO ( PUBYEAR , 2023 ) OR LIMIT-TO ( PUBYEAR , 2022 ) OR LIMIT-TO ( PUBYEAR , 2021 ) OR LIMIT-TO ( PUBYEAR , 2020 ) OR LIMIT-TO ( PUBYEAR , 2019 ) OR LIMIT-TO ( PUBYEAR , 2018 ) OR LIMIT-TO ( PUBYEAR , 2017 ) OR LIMIT-TO ( PUBYEAR , 2016 ) OR LIMIT-TO ( PUBYEAR , 2015 ) OR LIMIT-TO ( PUBYEAR , 2014 ) OR LIMIT-TO ( PUBYEAR , 2013 ) OR LIMIT-TO ( PUBYEAR , 2012 ) ) AND ( LIMIT-TO ( DOCTYPE , "ar" ) OR LIMIT-TO ( DOCTYPE , "re" ) ) AND ( LIMIT-TO ( LANGUAGE , "English" ) ) AND ( LIMIT-TO ( SRCTYPE , "j" ) )

#2: 5,217,299

TITLE-ABS-KEY ( "machine learning" ) OR TITLE-ABS-KEY ( "prediction model*" ) OR TITLE-ABS-KEY ( "neural network*" ) OR TITLE-ABS-KEY ( "deep learning" ) OR TITLE-ABS-KEY ( "artificial intelligence" ) OR TITLE-ABS-KEY ( ai ) OR TITLE-ABS-KEY ( "decision tree*" ) OR TITLE-ABS-KEY ( "computational intelligence" ) OR TITLE-ABS-KEY ( "machine intelligence" ) OR TITLE-ABS-KEY ( "algorithm*" ) OR TITLE-ABS-KEY ( "big data" ) OR TITLE-ABS-KEY ( bayesian ) OR TITLE-ABS-KEY ( "naïve bayes" ) OR TITLE-ABS-KEY ( "k-nearest neighbour" ) OR TITLE-ABS-KEY ( "decision support" ) OR TITLE-ABS-KEY ( "random forest" ) OR TITLE-ABS-KEY ( "support vector machine" ) OR TITLE-ABS-KEY ( svm ) OR TITLE-ABS-KEY ( xgboost ) OR TITLE-ABS-KEY ( adaboost ) OR TITLE-ABS-KEY ( "gradient boosting machine*" ) OR TITLE-ABS-KEY ( "regression tree*" ) OR TITLE-ABS-KEY ( "least squares" ) OR TITLE-ABS-KEY ( "stepwise regression" ) OR TITLE-ABS-KEY ( lstm ) OR TITLE-ABS-KEY ( rnn )

#3: 15,842,950

TITLE-ABS-KEY ( predict* ) OR TITLE-ABS-KEY ( detect* ) OR TITLE-ABS-KEY ( identif* ) OR TITLE-ABS-KEY ( "early diagnos*" )

#4: 7,916,598

TITLE-ABS-KEY ( trial* ) OR TITLE-ABS-KEY ( "clinical study" ) OR TITLE-ABS-KEY ( "prospective stud*" ) OR TITLE-ABS-KEY ( "pilot stud*" ) OR TITLE-ABS-KEY ( "controlled before-after stud*" ) OR TITLE-ABS-KEY ( "roll-out" ) OR TITLE-ABS-KEY ( "random controlled trial*" ) OR TITLE-ABS-KEY ( rct ) OR TITLE-ABS-KEY ( "controlled trial" ) OR TITLE-ABS-KEY ( "non-random* trial" ) OR TITLE-ABS-KEY ( prospective ) OR TITLE-ABS-KEY ( "prospective validation" ) OR TITLE-ABS-KEY ( implementation ) OR TITLE-ABS-KEY ( "pre-post implementation" )

#5: 3,156,593

( TITLE-ABS-KEY ( adolescen* ) OR TITLE-ABS-KEY ( child* ) OR TITLE-ABS-KEY ( schoolchild* ) OR TITLE-ABS-KEY ( infant* ) OR TITLE-ABS-KEY ( girl* ) OR TITLE-ABS-KEY ( boy* ) OR TITLE-ABS-KEY ( teen* ) OR TITLE-ABS-KEY ( youth* ) OR TITLE-ABS-KEY ( pediatr* ) OR TITLE-ABS-KEY ( paediatr* ) OR TITLE-ABS-KEY ( puber* ) ) AND NOT ( TITLE-ABS-KEY ( adult* ) OR TITLE-ABS-KEY ( man ) OR TITLE-ABS-KEY ( men ) OR TITLE-ABS-KEY ( woman ) OR TITLE-ABS-KEY ( women ) )

final: 1,482

#1 AND #2 AND #3 AND #4 AND NOT #5

( TITLE-ABS-KEY ( sepsis ) OR TITLE-ABS-KEY ( septic ) OR TITLE-ABS-KEY ( sirs ) OR TITLE-ABS-KEY ( "systemic inflammatory response syndrome" ) OR TITLE-ABS-KEY ( septicemia ) OR TITLE-ABS-KEY ( "sequential organ failure assessment" ) OR TITLE-ABS-KEY ( sofa ) OR TITLE-ABS-KEY ( qsofa ) ) AND ( TITLE-ABS-KEY ( "machine learning" ) OR TITLE-ABS-KEY ( "prediction model*" ) OR TITLE-ABS-KEY ( "neural network*" ) OR TITLE-ABS-KEY ( "deep learning" ) OR TITLE-ABS-KEY ( "artificial intelligence" ) OR TITLE-ABS-KEY ( ai ) OR TITLE-ABS-KEY ( "decision tree*" ) OR TITLE-ABS-KEY ( "computational intelligence" ) OR TITLE-ABS-KEY ( "machine intelligence" ) OR TITLE-ABS-KEY ( "algorithm*" ) OR TITLE-ABS-KEY ( "big data" ) OR TITLE-ABS-KEY ( bayesian ) OR TITLE-ABS-KEY ( "naïve bayes" ) OR TITLE-ABS-KEY ( "k-nearest neighbour" ) OR TITLE-ABS-KEY ( "decision support" ) OR TITLE-ABS-KEY ( "random forest" ) OR TITLE-ABS-KEY ( "support vector machine" ) OR TITLE-ABS-KEY ( svm ) OR TITLE-ABS-KEY ( xgboost ) OR TITLE-ABS-KEY ( adaboost ) OR TITLE-ABS-KEY ( "gradient boosting machine*" ) OR TITLE-ABS-KEY ( "regression tree*" ) OR TITLE-ABS-KEY ( "least squares" ) OR TITLE-ABS-KEY ( "stepwise regression" ) OR TITLE-ABS-KEY ( lstm ) OR TITLE-ABS-KEY ( rnn ) ) AND ( TITLE-ABS-KEY ( predict* ) OR TITLE-ABS-KEY ( detect* ) OR TITLE-ABS-KEY ( identif* ) OR TITLE-ABS-KEY ( "early diagnos*" ) ) AND ( TITLE-ABS-KEY ( trial* ) OR TITLE-ABS-KEY ( "clinical study" ) OR TITLE-ABS-KEY ( "prospective stud*" ) OR TITLE-ABS-KEY ( "pilot stud*" ) OR TITLE-ABS-KEY ( "controlled before-after stud*" ) OR TITLE-ABS-KEY ( "roll-out" ) OR TITLE-ABS-KEY ( "random controlled trial*" ) OR TITLE-ABS-KEY ( rct ) OR TITLE-ABS-KEY ( "controlled trial" ) OR TITLE-ABS-KEY ( "non-random* trial" ) OR TITLE-ABS-KEY ( prospective ) OR TITLE-ABS-KEY ( "prospective validation" ) OR TITLE-ABS-KEY ( implementation ) OR TITLE-ABS-KEY ( "pre-post implementation" ) ) AND NOT ( ( TITLE-ABS-KEY ( adolescen* ) OR TITLE-ABS-KEY ( child* ) OR TITLE-ABS-KEY ( schoolchild* ) OR TITLE-ABS-KEY ( infant* ) OR TITLE-ABS-KEY ( girl* ) OR TITLE-ABS-KEY ( boy* ) OR TITLE-ABS-KEY ( teen* ) OR TITLE-ABS-KEY ( youth* ) OR TITLE-ABS-KEY ( pediatr* ) OR TITLE-ABS-KEY ( paediatr* ) OR TITLE-ABS-KEY ( puber* ) ) AND NOT ( TITLE-ABS-KEY ( adult* ) OR TITLE-ABS-KEY ( man ) OR TITLE-ABS-KEY ( men ) OR TITLE-ABS-KEY ( woman ) OR TITLE-ABS-KEY ( women ) ) ) AND ( LIMIT-TO ( SRCTYPE , "j" ) ) AND ( LIMIT-TO ( DOCTYPE , "ar" ) OR LIMIT-TO ( DOCTYPE , "re" ) ) AND ( LIMIT-TO ( PUBYEAR , 2024 ) OR LIMIT-TO ( PUBYEAR , 2023 ) OR LIMIT-TO ( PUBYEAR , 2022 ) OR LIMIT-TO ( PUBYEAR , 2021 ) OR LIMIT-TO ( PUBYEAR , 2020 ) OR LIMIT-TO ( PUBYEAR , 2019 ) OR LIMIT-TO ( PUBYEAR , 2018 ) OR LIMIT-TO ( PUBYEAR , 2017 ) OR LIMIT-TO ( PUBYEAR , 2016 ) OR LIMIT-TO ( PUBYEAR , 2015 ) OR LIMIT-TO ( PUBYEAR , 2014 ) OR LIMIT-TO ( PUBYEAR , 2013 ) OR LIMIT-TO ( PUBYEAR , 2012 ) ) AND ( LIMIT-TO ( LANGUAGE , "English" ) )

## Web of Science search details

#11 (#2 AND #3 AND #4 AND #5) NOT #6 and 2022 or 2021 or 2020 or 2019 or 2018 or 2017 or 2016 or 2015 or 2014 or 2013 or 2012 (Publication Years) and Articles or Review Articles or Early Access (Document Types) and English (Languages)

#2: (TI= (sepsis OR septic OR sirs OR "systemic inflammatory response syndrome" OR septicemia OR "sequential organ failure assessment" OR sofa OR qsofa)) OR (AB= (sepsis OR septic OR sirs OR "systemic inflammatory response syndrome" OR septicemia OR "sequential organ failure assessment" OR sofa OR qsofa)) OR (AK= (sepsis OR septic OR sirs OR "systemic inflammatory response syndrome" OR septicemia OR "sequential organ failure assessment" OR sofa OR qsofa))

#3 (TI=("machine learning" OR "prediction model*" OR "neural network*" OR "deep learning" OR "artificial intelligence" OR ai OR "decision tree*" OR "computational intelligence" OR "machine intelligence" OR "algorithm*" OR "big data" OR bayesian OR "naïve bayes" OR "k-nearest neighbour" OR "decision support" OR "random forest" OR "support vector machine" OR svm OR xgboost OR adaboost OR "gradient boosting machine*" OR "regression tree*" OR "least squares" OR "stepwise regression" OR lstm OR rnn)) or (AB=("machine learning" OR "prediction model*" OR "neural network*" OR "deep learning" OR "artificial intelligence" OR ai OR "decision tree*" OR "computational intelligence" OR "machine intelligence" OR "algorithm*" OR "big data" OR bayesian OR "naïve bayes" OR "k-nearest neighbour" OR "decision support" OR "random forest" OR "support vector machine" OR svm OR xgboost OR adaboost OR "gradient boosting machine*" OR "regression tree*" OR "least squares" OR "stepwise regression" OR lstm OR rnn)) OR (AK=("machine learning" OR "prediction model*" OR "neural network*" OR "deep learning" OR "artificial intelligence" OR ai OR "decision tree*" OR "computational intelligence" OR "machine intelligence" OR "algorithm*" OR "big data" OR bayesian OR "naïve bayes" OR "k-nearest neighbour" OR "decision support" OR "random forest" OR "support vector machine" OR svm OR xgboost OR adaboost OR "gradient boosting machine*" OR "regression tree*" OR "least squares" OR "stepwise regression" OR lstm OR rnn))

#4: (TI=(predict* OR detect* OR identif* OR "early diagnos*")) OR (AB=(predict* OR detect* OR identif* OR "early diagnos*")) OR (AK=(predict* OR detect* OR identif* OR "early diagnos*"))

#5 (TI=(trial* OR "clinical study" OR "prospective stud*" OR "pilot stud*" OR "controlled before-after stud*" OR "roll-out" OR "random controlled trial*" OR rct OR "controlled trial" OR "non-random* trial" OR prospective OR "prospective validation" OR implementation OR "pre-post implementation")) OR (AB=(trial* OR "clinical study" OR "prospective stud*" OR "pilot stud*" OR "controlled before-after stud*" OR "roll-out" OR "random controlled trial*" OR rct OR "controlled trial" OR "non-random* trial" OR prospective OR "prospective validation" OR implementation OR "pre-post implementation")) OR (AK=(trial* OR "clinical study" OR "prospective stud*" OR "pilot stud*" OR "controlled before-after stud*" OR "roll-out" OR "random controlled trial*" OR rct OR "controlled trial" OR "non-random* trial" OR prospective OR "prospective validation" OR implementation OR "pre-post implementation"))

#6 ((TI=(adolescen* OR child* OR schoolchild* OR infant* OR girl* OR boy* OR teen* OR youth* OR pediatr* OR paediatr* OR puber*)) OR (AB=(adolescen* OR child* OR schoolchild* OR infant* OR girl* OR boy* OR teen* OR youth* OR pediatr* OR paediatr* OR puber*)) OR (AK=(adolescen* OR child* OR schoolchild* OR infant* OR girl* OR boy* OR teen* OR youth* OR pediatr* OR paediatr* OR puber*))) NOT ((TI=(adult* OR man OR men OR woman OR women)) OR (AB=(adult* OR man OR men OR woman OR women)) OR (AK=(adult* OR man OR men OR woman OR women)))

The query result numbers were missing for this search and so a screen-shot was taken (see Figure A1 below) of the final screen with the final query result number (341).


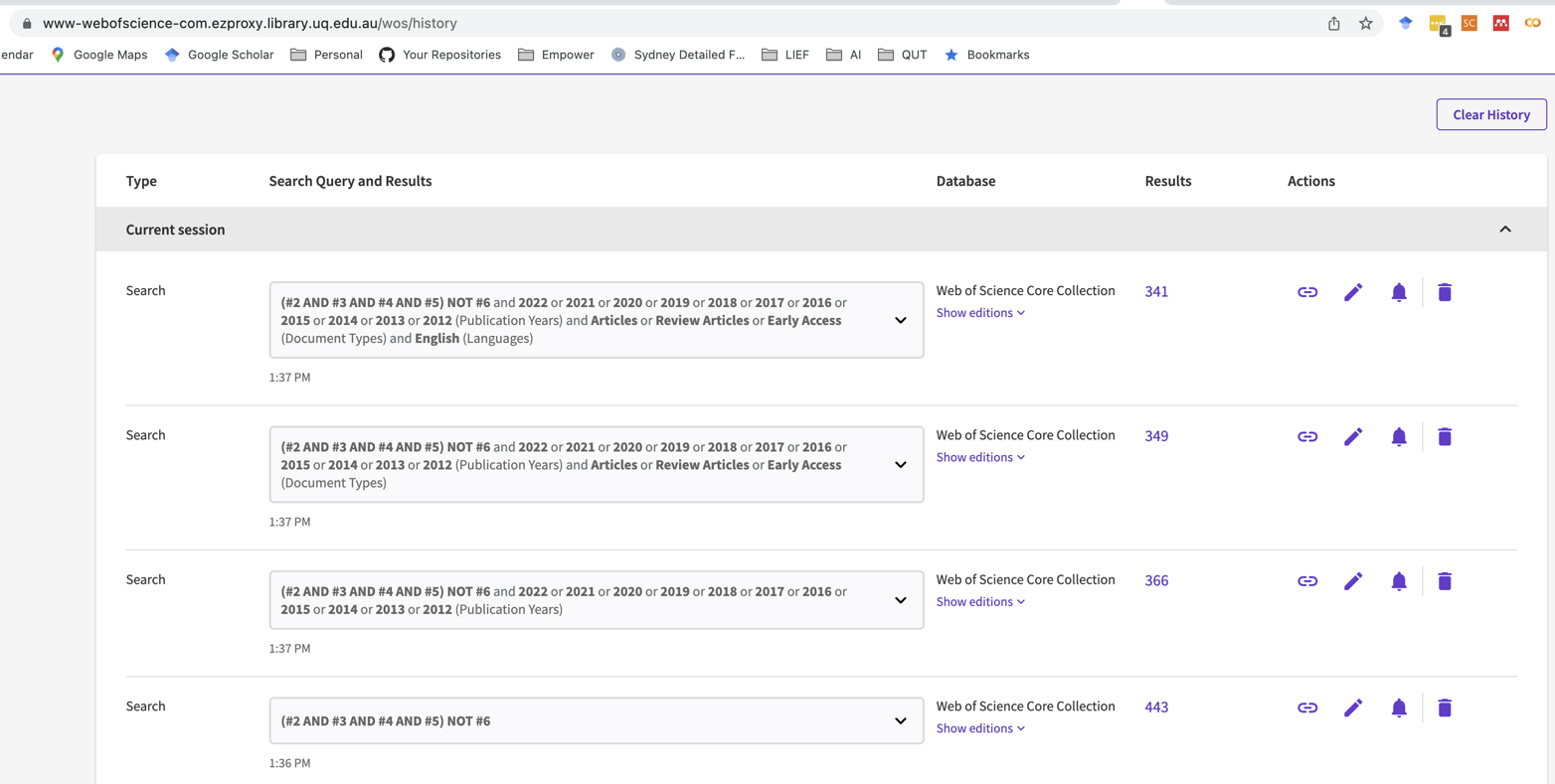


Figure A1: Web of science screenshot of final query numbers.

# Appendix B. Selection criteria


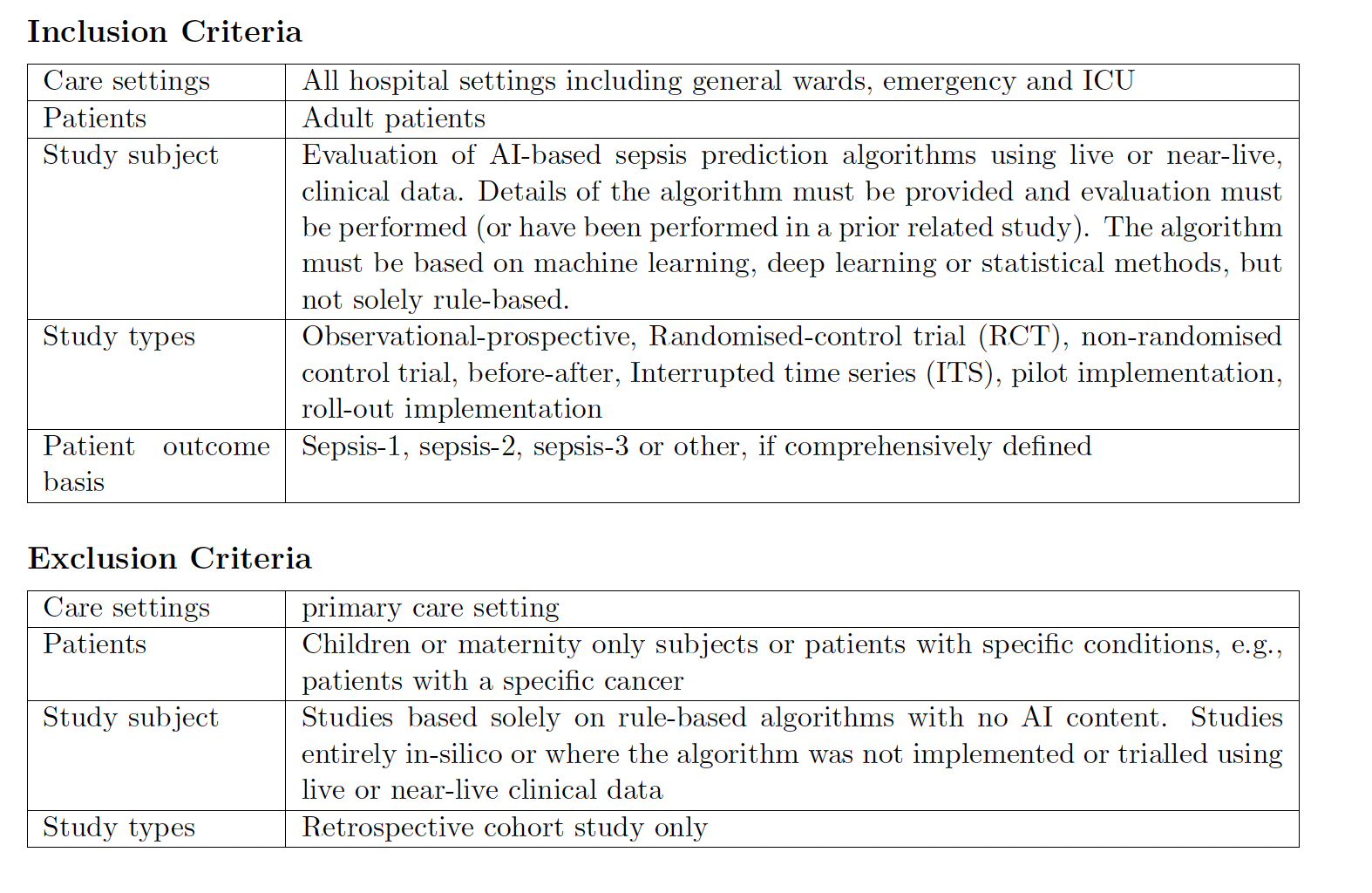


†

| †  SIRS definition: Sepsis 1 (1991)  REF: Bone RC, Balk RA, Cerra FB, et al. Definitions for sepsis and organ failure and guidelines for the use of innovative therapies in sepsis. Chest 1992;101:1644–55 |
| --- |
| SIRS definition: Sepsis 2 (2001)  REF: Levy MM, Fink MP, Marshall JC, et al., 2001 sccm/esicm/accp/ats/sis in-ternational sepsis definitions conference, Intensive Care Med. 29 (4) (2003Apr 1) 530e538 |
| SIRS definition: Sepsis 3 (2016)  REF: Singer M, Deutschman CS, Seymour CW, et al. The Third International Consensus Definitions for Sepsis and Septic Shock (Sepsis-3). JAMA 2016 Feb 23;315(8):801-810. |

# Appendix C. Extracted data items

## Paper Information

| Year | Journal | Country |
| --- | --- | --- |
| Study purpose/aim | Study design | Implementation type: [specific\|general] |
| Specific details [optional] | MLA name | Standard reporting framework used |

## Retrospective dataset details

| Care setting | Care setting detail | Number of centres |
| --- | --- | --- |
| Centre descriptions | Number of beds | Annual admissions |
| Linkage to prior MLA study | Dataset details | Patient number |
| Sepsis outcome criteria | Outcome number | Prevalence |
| Inclusion/exclusion criteria | Algorithm performance and basis | Comparison model performance and basis |
| ML algorithm description | ML algorithm used | ML hours of data required before prediction can occur |
| ML number of features | Was feature/variable importance reported | Data pipeline/infrastructure details |

## Validation dataset details

| Care setting detail | Dataset inclusion/exclusion details | Patient number |
| --- | --- | --- |
| Sepsis outcome criteria | Outcome number | Prevalence |
| How was algorithm setpoint/cut-off set? | Algorithm performance | Comparison results |
| Concerns related to validation |  |  |

## Implementation details

| Small (<100 patients) or large-scale trial | Organisation/group responsible for the implementation and timeframe | Implementation process followed (if any) |
| --- | --- | --- |
| Site details | Patient/encounter numbers | Dataset details |
| Patient inclusions/exclusions | Data sampling details | How was algorithm setpoint/cut-off set |
| If randomized, how? | If blinding, how? | Clinician training description |
| Control care provided | Intervention care provided | Control care vs implementation care |
| Sepsis outcome criteria used | Outcome number | Sepsis prevalence |
| Concerns related to implementation | Did silent/shadow trial/ | Shadow trial patient number |
| Trial description | Description of timing of alerts | Alert frequency method, e.g., one-time-only |
| Details on development of HCI/alert | Where was alert sent and by what means | Required actions based on alert |
| Actual response times to alert | How did evaluation consider whether clinicians already suspected sepsis | Results: alert timing vs onset of sepsis |
| Results: algorithm performance | Possible implementation measures/evaluation used | Summary of positive clinical process results |
| Summary of negative/null clinical process results | Summary of positive clinical outcome results | Summary of null/negative clinical outcome results |
| Overall conclusion regarding impact on clinical outcomes | Overall conclusion regarding implementation and capability of algorithm pipeline | Reported study limitations |
| Conflicts of interest | Barriers | Enablers |
| Key decisions |  |  |

# Appendix D: Risk of Bias Assessment

**Table D1: Risk of bias (ROB) assessment of studies reporting mortality**

|  |  | **Confounding** | **Selection of participants (Randomization for RCT)** | **Classification of interventions** | **Deviation from intended interventions** | **Missing data** | **Measurement of outcomes** | **Selection of reported results** | **Overall** |
| --- | --- | --- | --- | --- | --- | --- | --- | --- | --- |
| **Group A (EWS2.0)** | |  |  |  |  |  |  |  |  |
|  | Gianinni et al, 2019 | Serious | Low | Low | Low | Low | Low | Moderate | Serious |
| **Group B (Insight)** | |  |  |  |  |  |  |  |  |
|  | ^ꝉ^ McCoy et al., 2017 | Critical | Low | Moderate | Serious | Low | Moderate | Moderate | Critical |
|  | ^ꝉ^ Burdick et al, 2018 | Serious | Low | Low | Low | Low | Moderate | Moderate | Serious |
|  | Burdick et al, 2020 | Serious | Serious | Low | Moderate | Low | Moderate | Moderate | Serious |
|  | Shimabukuro, 2017 |  | Low |  | Low | Low | Low | Some concerns | Some concerns |
| **Group C (Robot Laura)** | |  |  |  |  |  |  |  |  |
|  | No suitable trial for evaluation of mortality | | | | |  |  |  |  |
| **Group D (Sepsis Watch)** | |  |  |  |  |  |  |  |  |
|  | No suitable trial for evaluation of mortality | | | | |  |  |  |  |
| **Group E (TREWScore)** | |  |  |  |  |  |  |  |  |
|  | Adams et al, 2022 | Moderate | Low | Low | Low | Low | Moderate | Low | Moderate |
| **Group F (Sepsis Sniffer)** | |  |  |  |  |  |  |  |  |
|  | Lipatov et al, 2022 | Serious | Low | Low | Low | Low | Low | Low | Serious |
| **Group G (ESM)** | |  |  |  |  |  |  |  |  |
|  | Schootman et al, 2022 | Moderate | Low | Low | Low | Low | Low | Low | Moderate |
| **Group H (No name)** | |  |  |  |  |  |  |  |  |
|  | No suitable trial for mortality outcome | | | | |  |  |  |  |

All studies are assessed using ROBINS-I tool^24^ for non-randomised studies, except for Shimabukuro, which is assessed using the Cochrane Risk-of-bias-2 tool for randomized studies.^25^ ꝉ indicates sepsis-related mortality; all other studies reported all-cause mortality. The bias scale is: Low risk of bias (the study is comparable to a well-performed randomized trial with regard to this domain); Moderate risk of bias (the study is sound for a non-randomized study with regard to this domain but cannot be considered comparable to a well-performed randomized trial); Serious risk of bias (the study has some important problems); Critical risk of bias (the study is too problematic to provide any useful evidence on the effects of intervention).

# Appendix E: Extracted Evaluation Metrics

| **Metric Number** | **Count Papers** | **% Coverage** | **Implementation Groups** | | **Metric** |
| --- | --- | --- | --- | --- | --- |
|  |  |  |  | | **Model Performance** |
| 1 | 17 | 65% | 6 | | Area under the receiver operating cuve (AUC) |
| 2 | 3 | 12% | 2 | | Area under precision-recall curves (APR) |
| 3 | 2 | 8% | 1 | | Accuracy (%) |
| 4 | 3 | 12% | 2 | | False positive rate |
| 5 | 1 | 4% | 1 | | False negative rate |
| 6 | 18 | 69% | 7 | | Sensitivity (%) |
| 7 | 13 | 50% | 6 | | Specificity (%) |
| 8 | 2 | 8% | 1 | | F1 (%) |
| 9 | 9 | 35% | 7 | | PPV (%) |
| 10 | 6 | 23% | 4 | | NPV (%) |
| 11 | 3 | 12% | 2 | | Positive likelihood ratio |
| 12 | 3 | 12% | 2 | | Negative likelihood ratio |
| 13 | 2 | 8% | 1 | | Diagnostic odds ratio |
| 14 | 1 | 4% | 1 | | Number of evaluations needed to identity a single patient with eventual sepsis |
| 15 | 1 | 4% | 1 | | Reduction in false alarms |
| 16 | 1 | 4% | 1 | | False negative count |
| 17 | 1 | 4% | 1 | | Median false positive per day |
| 18 | 3 | 12% | 2 | | False alarm : True alarm |
| 19 | 3 | 12% | 3 | | Lead time to onset |
| 20 | 2 | 8% | 2 | | % Alert of SOFA progression |
| 21 | 4 | 15% | 4 | | % Alerts undetected by clinicians / other tools |
| 22 | 1 | 4% | 1 | | Duration of delay vs clinical suspicion for true positives |
| 23 | 1 | 4% | 1 | | Duration of delay vs antibiotic administration for true positives |
| 24 | 1 | 4% | 1 | | % alerts received for true positive patients already on antibiotics |
| 25 | 1 | 4% | 1 | | % alerts received for patients already in ICU |
| 26 | 5 | 19% | 4 | | % Alerted patients in the data |
|  | 120 |  |  | |  |
|  |  |  |  | | **Clinical Patient Outcomes** |
| 1 | 9 | 35% | 5 | | Decrease in mortality |
| 2 | 2 | 8% | 2 | | Odds ratio for in-hospital mortality per hour delay in antibiotics |
| 3 | 9 | 35% | 5 | | Decrease in length of stay |
| 4 | 2 | 8% | 2 | | Improved SOFA score progression |
| 5 | 1 | 4% | 1 | | Improved acute physiology and chronic health evaluation III score |
| 6 | 2 | 8% | 1 | | Reduction in readmission rate |
| 7 | 1 | 4% | 1 | | Improved discharge disposition |
| 8 | 1 | 4% | 1 | | Antibiotic lead time to mortality |
| 9 | 1 | 4% | 1 | | Antibiotic lead time to SOFA progression |
| 10 | 1 | 4% | 1 | | Antibiotic lead time -> median LOS (hours) |
|  |  |  |  | |  |
|  |  |  |  | | **Clinical Process Outcomes** |
| 11 | 5 | 19% | 4 | | Median lead time from alert to first antibiotic (hours) |
| 12 | 1 | 4% | 1 | | Time to fluid resuscitation |
| Mention, not used | | | | Time from ED presentation to sepsis onset | |
| Mention, not used | | | | Time from sepsis onset to bundle completion | |
| 13 | 1 | 4% | 1 | | % rate of antibiotic <1h |
| 14 | 3 | 12% | 3 | | % 3-H bundle compliance rate |
| 15 | 3 | 12% | 3 | | % 3-H antibiotic compliance |
| 16 | 1 | 4% | 1 | | % 3-H serum lactate / fluid bolus compliance |
| 17 | 1 | 4% | 1 | | % 3-H blood culture compliance |
| 18 | 1 | 4% | 1 | | SBP normalization within 15 min |
| 19 | 1 | 4% | 1 | | MAP normalization within 15 min |
| 20 | 1 | 4% | 1 | | Lactate normalization time, minutes |
| 21 | 1 | 4% | 1 | | Vital sign EMR entry delay 15min-1hr (%) |
| 22 | 1 | 4% | 1 | | Laboratory values EMR entry delay 15min-1hr (%) |
| 23 | 1 | 4% | 1 | | % No lactate measurement |
| 24 | 1 | 4% | 1 | | % No CVP measurement |
| 25 | 1 | 4% | 1 | | % Delay (no lactate + CVP) |
| 26 | 1 | 4% | 1 | | Increase in bundle administration |
| 27 | 3 | 12% | 3 | | Increase in antibiotic use |
| 28 | 1 | 4% | 1 | | Increase in lactate testing |
| 29 | 2 | 8% | 2 | | Increase in IV fluid |
| 30 | 1 | 4% | 1 | | Increase in blood tests |
| 31 | 1 | 4% | 1 | | Increase in RBC transfusion |
| 32 | 1 | 4% | 1 | | Increase in blood cultures |
| 33 | 1 | 4% | 1 | | Increase in telemetry or electrocardiogram |
| 34 | 1 | 4% | 1 | | Increase in chest radiograph |
| 35 | 1 | 4% | 1 | | Increase in CT imaging |
| 36 | 1 | 4% | 1 | | Increase in diuretic |
| 37 | 1 | 4% | 1 | | Increase in atrioventricular nodal blockade |
| 38 | 1 | 4% | 1 | | Increase in arterial blood gas |
| 39 | 1 | 4% | 1 | | Increase in vasopressors |
| 40 | 1 | 4% | 1 | | Increase in naloxone |
| Mention, not used | | | | Antibiotics overtreatment | |
| 41 | 1 | 4% | 1 | | % No severe sepsis CPOE (computerized physician order entry) |
| 42 | 1 | 4% | 1 | | % Delay + CPOE (overlap) |
| 43 | 1 | 4% | 1 | | Decrease in development of severe sepsis / septic shock |
| 44 | 1 | 4% | 1 | | Decrease in time to ICU transfer |
| 45 | 2 | 8% | 2 | | Decrease in frequency of ICU transfer |
| 46 | 1 | 4% | 1 | | Increase in discharge to home/inpatient hospice/nursing facility |
| Mention, not used | | | | Average number of new patients alerted per day | |
| Mention, not used | | | | Distribution of new patients alerted by hour | |
| Mention, not used | | | | Average number of patients treated with sepsis bundle per day | |
|  |  |  |  | |  |
| 1 | 2 | 8% | 1 | | **Financial Impact** |
|  |  |  |  | |  |
|  |  |  |  | | **Adoption Metrics:** |
| 1 | 4 | 15% | 3 | | % Alert evaluation |
| 2 | 1 | 4% | 1 | | % Retrospective sepsis cases with alerts evaluated |
| 3 | 5 | 19% | 3 | | % Alerts confirmed (sepsis) |
| 4 | 1 | 4% | 1 | | % Retrospective sepsis cases with alerts confirmed |

Table E1: All metrics used across the reviewed studies. Metrics are grouped into Model performance, clinical patient outcomes, clinical process outcomes, financial impact and adoption metrics. Implementation groups specifies the number of MLA groups that used the metric whereas column (2), count papers, identifies the number of individual studies that use each metric. The coverage is based on the number of papers using the metric divided by the total number of papers that report quantifiable data (n=26)

# Appendix F: Sepsis definitions employed by each included and linked study

## Table F1: Criteria utilised by papers, together with references, if provided

| **Criteria ID** | **Criteria specified (with references where applicable)** |
| --- | --- |
| A | ICD-9: 995.92 - severe sepsis |
| B | ICD-9: 785.52 (septic shock) |
| C | A positive blood culture |
| D | lactate > than 2.2 mmol/L |
| E | systolic blood pressure < 90 mm HG |
| F | two or more SIRS (unspecified) criteria |
| G | A positive urine culture |
| H | ICD-9: 995.9: in-hospital sepsis |
| I | SIRS definition: Sepsis 1 (1991)  REF: Bone RC, Balk RA, Cerra FB, et al. Definitions for sepsis and organ failure and guidelines for the use of innovative therapies in sepsis. Chest 1992;101:1644–55 |
| J | SIRS definition: Sepsis 2 (2001)  REF: Levy MM, Fink MP, Marshall JC, et al., 2001 sccm/esicm/accp/ats/sis in-ternational sepsis definitions conference, Intensive Care Med. 29 (4) (2003Apr 1) 530e538 |
| K | SIRS definition: Sepsis 3 (2016)  REF: Singer M, Deutschman CS, Seymour CW, et al. The Third International Consensus Definitions for Sepsis and Septic Shock (Sepsis-3). JAMA 2016 Feb 23;315(8):801-810. |
| L | infection-related ICD-9 code |
| M | organ disfunction |
| N | total fluid replacement >=1200 mL or >=20 mL/kg |
| O | suspicion of infection = order for a culture lab draw + a dose of antibiotics, within a specified window |
| P | acute change in total SOFA score ≥2 points |
| Q | ICD-10: R65 (sepsis) |
| R | ICD-10: R652 (Severe sepsis) |
| S | ICD-10: R6521 (Septic shock) |
| T | Two or more specified SIRS criteria |
| U | ICD-9: 995.91 (sepsis) |
| V | ICD-10: 29 specific sepsis codes |
| W | at least two consistently abnormal vitals signs |
| X | Blood culture drawn |
| Y | at least one abnormal laboratory value in-dicating early signs of organ failure |
| Z | organ disfunction caused by sepsis (SSC guidelines)  REF: Dellinger RP, Levy MM, Rhodes A, et al; Surviving Sepsis Campaign Guidelines Committee including the Pediatric Subgroup, Surviving Sepsis Campaign: International guidelines for management of severe sepsis and septic shock: 2012.Crit. Care Med.41,580–637 (2013) |
| AA | Suspicion of infection: ICD-9 codes  REF: Angus C, Linde-Zwirble WT, Lidicker J, et al. Epidemiology of severe sepsis in the United States: Analysis of incidence, outcome, and associated costs of care. Crit Care Med.2001; 29, 1303–1310. |
| AB | clinical note that mentioned sepsis or septic shock. |
| AC | patients with features consistent with sepsis, but explained by other conditions (for example, hemorrhage) are excluded.  REF: Henry KE, Hager DN, Osborn T, et al. Comparison of automated sepsis identification methods and electronic health record-based sepsis phenotyping: improving case identification accuracy by accounting for confounding comorbid conditions. Crit Care Explor 2019; 1: e0053. |
| AD | Suspicion of infection: Any culture order, e.g. blood, urine, stool, lavage, sputum |
| AE | Organ hypoperfusion and dysfunction: Lactate >=4 mmol/L OR SBP <=90 mm Hg |
| AF | vasopressors administered: Norepinephrine, epinephrine, dopamine, vasopressin, or phenylephrine |
| AG | Fluid resistant hypotension: BP<90 mm Hg despite >=30 mL/kg crystalloid and/or 18.75 mL/kg colloid fluid bolus |
| AH | SBP < 90 mm Hg despite≥30 ml/kg fluid bolus |
| AI | the Centers for Disease Control and Prevention clinical surveillance definition |
| AJ | Centers for Medicare & Medicaid Services criterion for organ dysfunction |
| AK | CD-10-CM Principal or Other Diagnosis Code of Sepsis, Severe Sepsis, or Septic Shock are included (A02.1, A22.7, A26.7, A32.7, A40.0, A40.1, A40.3, A40.8, A40.9,A41.01, A41.02, A41.1, A41.2, A41.3, A41.4, A41.50, A41.51, A41.52,A41.53, A41.59, A41.81, A41.89, A41.9, A42.7, A54.86, B37.7, R65.20,R65.21) |
| AL | ICD-9 codes were chosen to include broad representation of diagnoses associated with sepsis or infection |

##

## Table F2: Sepsis definitions used by each paper

The ID is the identifier of the definition referenced in the paper (Table 3). The name is the sepsis name stated by the paper. The prediction target is the actual targeted sepsis level, i.e. sepsis, sever sepsis or septic shock. The logic provides the criteria used, from Criteria in Table G1, and logical operators needed to define the sepsis definition

| **ID** | **Name** | **Prediction-target** | **Logic** | **Study IDs** |
| --- | --- | --- | --- | --- |
| [a] | Sepsis training criteria | sepsis/Severe | {A OR B AND C AND (D OR E)} - within a 1hr window | Giannini et al, 2019 |
| [b] | Severe sepsis | Severe | {F AND D AND (G OR C)} within 1hr | Giannini et al, 2019 |
| [c] | Septic shock | Shock | {[b] AND E} within 1hr | Giannini et al, 2019 |
| [d] | in-hospital sepsis (IHAS) | sepsis | H AND {I for 5hrs} | Calvert et al, 2016 |
| [e] | Septic shock | Shock | {J criteria >=2} AND L And M AND {E 1hr min} AND {N for 24hrs} | Calvert et al, 2016b |
| [f] | Sepsis-3 sepsis | sepsis | K impl as O AND P in timeframe | Desautels et al, 2016 |
| [g) | Sepsis | sepsis | Q | Shimabukuro et al, 2017 |
| [h] | Severe sepsis | Severe | R | Shimabukuro et al, 2017 |
| [i] | Septic shock | Shock | S | Shimabukuro et al, 2017 |
| [j] | Sepsis | sepsis | {T based on I) at some point during hospital stay | McCoy et al, 2017 Burdick et al., 2018 Burdick et al., 2020 |
| [k] | ALL | Sep/Sev/Sho | A or B or U | Mao et al, 2017 |
| [l] | Any kind of sepsis | ALL | V | Topiwala et al., 2019 |
| [m] | Suspected sepsis | sepsis | W AND X AND Y | Futoma et al., 2017 Futoma et al., 2017b |
| [n] | Sepsis | sepsis | F AND X AND Y | Bedoya et al., 2020 |
| [o] | sepsis | sepsis | F AND (AA Or AB) | Henry et al., 2015 |
| [p] | Severe sepsis | Severe | [o] AND Z | Henry et al., 2015 |
| [q] | Septic shock | Shock | [p] AND {E >= 30 mins} AND {N past 24h} | Henry et al., 2015 |
| [r] | EHR-based sepsis phenotyping | sepsis | K AND NOT AC | Adams et al, 2022 Henry et al, 2022 |
| [s] | Severe sepsis | Severe | {AD AND I AND AE} within 6hrs | Harrison et al, 2015 |
| [t] | Septic shock | Shock | [s] AND {AF OR AG within 3hr} | Harrison et al, 2015 |
| [u] | sepsis | sepsis | T within 6hr window | Lipatov et al., 2022 |
| [v] | Severe sepsis | severe | [u] + AE | Lipatov et al., 2022 |
| [w] | Septic shock | Shock | [v] + {AF OR AH} within 3hr | Lipatov et al., 2022 |
| [x] | sepsis | sepsis | AI OR (Q AND F AND AJ) within 6hrs | Wong et al., 2021 |
| [y] | severe or shock | severe/shock | AK | Schootman et al., 2022 |
| [z] | Severe or shock | Severe/shock | I AND AL | Brown et al., 2016 |
